# Development of Cell Technologies Based on Dendritic Cells for Immunotherapy of Oncological Diseases

**DOI:** 10.3390/biomedicines12030699

**Published:** 2024-03-21

**Authors:** Vasily Kurilin, Alina Alshevskaya, Sergey Sennikov

**Affiliations:** 1 Laboratory of Molecular Immunology, Federal State Budgetary Scientific Institution “Research Institute of Fundamental and Clinical Immunology” (RIFCI), 630099 Novosibirsk, Russia; vkurilin@niikim.ru; 2 Laboratory of Immuno Engineering, Federal State Autonomous Educational Institution of Higher Education I.M. Sechenov First Moscow State Medical University of the Ministry of Health of the Russian Federation (Sechenov University), 119048 Moscow, Russia; alshevskaya_a_a@staff.sechenov.ru

**Keywords:** dendritic cells, tumor antigens, immune response, cell technologies, tumor immunotherapy

## Abstract

Immunotherapy using dendritic cell-based vaccination is a natural approach using the capabilities and functions inherent in the patient’s immune system to eliminate tumor cells. The development of dendritic cell-based cell technologies evolved as the disorders of dendritic cell differentiation and function in cancer were studied; some of these functions are antigen presentation, priming of cytotoxic T-lymphocytes and induction of antigen-specific immune responses. At the initial stage of technology development, it was necessary to develop protocols for the in vitro generation of functionally mature dendritic cells that were capable of capturing tumor antigens and processing and presenting them in complex with MHC to T-lymphocytes. To achieve this, various forms of tumor-associated antigen delivery systems were tested, including lysates, tumor cell proteins (peptides), and DNA and RNA constructs, and it was shown that the use of DNA and RNA constructs was the most effective method, as it made it possible not only to deliver the most immunogenic epitopes of tumor-associated antigens to dendritic cells, but also to enhance their ability to induce antigen-specific cytotoxic T-lymphocytes. Currently, cell therapy based on dendritic cells is a modern basis for antigen-specific immunotherapy of cancer due to the simplicity of creating DNA and RNA constructs encoding information about both target tumor antigens and regulatory molecules. The potential development of cell technologies based on dendritic cells aims to obtain antigen-specific cytotoxic T-lymphocytes induced by dendritic cells, study their functional activity and develop cell-based therapy.

## 1. Introduction

Oncological diseases are some of the leading causes of death. The high incidence of cancer can be explained by various factors (for example, adverse environmental conditions, social stress and the increased incidence of viral infections). As our understanding of the immune system and its functions expanded, it became apparent that the general reactivity of the immune system undergoes shifts during the course of human development. On the one hand, there is the involution of the thymus, and, on the other hand, there is the “training” of the immune system through targeted vaccination and as a result of transferred diseases. All these processes can influence the activity of the immune system in general and the antitumor immune response in particular. The key players in the activation of antigen-specific immune responses are antigen-presenting cells, dendritic cells in particular, which mediate antitumor immunity through the activation of CD8+ and CD4+ T-cells in the tumor microenvironment. The defects of dendritic cell maturation and differentiation described in cancer patients are associated with ineffective antitumor defense. Therefore, significant attention is directed towards the restoration of the antigen-presenting function of dendritic cells, which, in turn, is correlated with a more effective antitumor immune response. Functionally mature dendritic cells were obtained under in vitro conditions using various protocols for their generation from monocytes of cancer patients, with subsequent “training” by delivery of tumor antigens in the form of peptides, lysates, DNA plasmids or RNA by various methods (liposomes, transfection and transduction). Protocols for the induction of antitumor immune responses using induced antigen-specific dendritic cells showed their efficacy in both in vitro and in vivo studies, including clinical studies.

## 2. Disruption of Induction and Development of Antitumor Immune Response in the Cancer Process

Tumors contain heterogeneous cancer cells, including tumor stem cells [1,2], which interact with microenvironmental cells and immune cells [1,2]. Progressive tumor growth is associated with escape from immune control [3]. The development of the tumor process is associated with the impact of malignant cells and their microenvironment on immune cells, causing their suppression and changes in their activity. It has been shown that in patients with cancer, the number of circulating dendritic cells, in particular myeloid dendritic cells, decreases, and the ratio of myeloid and plasmacytoid dendritic cells changes. These changes are indicated for patients with breast cancer, melanoma and pancreatic cancer [4,5,6]. In addition to changes in the number and ratio of the main populations of dendritic cells, there is also a violation of their maturation and differentiation and suppression of their functional activity [7,8]. Reduced accumulation of mature dendritic cells was found in patients with cervical cancer [9], hepatocellular carcinoma [10], lung cancer [11], colorectal cancer [12] and breast cancer [13]. Blockade of dendritic cell differentiation in cancer patients is most often associated with VEGF, a tumor microenvironmental factor that stimulates tumor angiogenesis [14,15]. Accordingly, the content of VEGF negatively correlates with the number of dendritic cells in the blood and tumors in various types of human cancer [16]. These studies suggest that the lack of functionally mature dendritic cells in tumors is a major factor contributing to overall immune evasion. A consequence of defective antigen presentation in cancer patients is a lack of T cell response, which may result from anergy or exhaustion. It should be noted that anergy and exhaustion are reversible processes that arise as a result of different transcriptional programs [17,18]. Anergy occurs during priming, whereas exhaustion occurs in previously activated T cells that undergo repeated exposure to suboptimal amounts of antigens in the presence of negative costimulation. In this regard, T cell exhaustion has been shown to be a major factor in tumor-induced immunosuppression in a number of cancer patients [19,20]. The negative influence of tumor cells and their microenvironment is manifested in the differentiation of effector cells and dendritic cells into suppressor cells [21], mediated by “checkpoint” molecules, which is highly expressed in tumor-infiltrating dendritic cells, inhibiting T-cell activation and cytokine production [22]. Another mechanism involves enhancing the activity of T-cell immunoglobulin and mucin domain-containing protein-3 (TIM-3) on dendritic cells, which inhibits immune signal transduction [23,24]. The presence of VEGF, CCL1, CCL2 and CXCL5 in a conditioned medium from colorectal cancer explants was shown to inhibit dendritic cell maturation and IL-12 production, while IL-10 secretion increased [25]. Melanoma cells expressing β-catenin were found to induce resistance to immunotherapeutic agents, reduce dendritic cell and T-cell infiltration, and promote tumor growth [26]. Moreover, the presence of prostaglandin E2 stimulated tumor growth by disrupting the accumulation of intratumoral CD103+ dendritic cells [27]. Metabolic dysfunction may also affect dendritic cell maturation in cancer patients. Hypoxia, lactic acid production and decreased pH impair normal dendritic cell function [28]. Metabolites from the tumor microenvironment have been shown to induce lipid peroxidation, which ultimately leads to lipid accumulation in dendritic cells [29,30]. These lipid particles inhibit the migration of the peptide–MHC I complex to the surface of dendritic cells and impair the cross-presentation potential of T cells, blocking their activity [31,32]. It has been shown that tumor-infiltrating plasmacytoid dendritic cells are not capable of producing type I IFN but actively express indoleamine 2,3-dioxygenase (IDO) and ICOSL and stimulate Treg cells. This enhances tumor progression [33,34]. A large number of tumor-infiltrating plasmacytoid dendritic cells in cancer patients is associated with a negative prognosis [35,36]. Dendritic cells in the tumor microenvironment have a lower antigen trafficking potential due to controlled expression of CCR7 [37], resulting in a reduced ability to induce T cells in lymph nodes.

## 3. Dendritic Cells, Tumor Antigen Presentation and Induction of Antitumor Immune Response

Antigen-presenting cells (macrophages, dendritic cells and B cells) play an important role in both the innate and adaptive immune responses [38]. Dendritic cells are the most important of the antigen-presenting cells, which were first described by Ralph Steinman in 1973. They perform a number of specific functions in immunological processes, such as the initiation, regulation and maintenance of immune responses [39].

Currently, the heterogeneity of dendritic cells, their division into subgroups with different phenotypic and functional properties, and their role in immunoregulation have been described [40,41]. Four major subpopulations of dendritic cells involved in antitumor immunity have been described. The first two are represented by subgroups of conventional (myeloid) dendritic cells (cDCs): CD11c+ MHC class II+ cDCs, which are present in peripheral and lymphoid tissues: cDCs type 1 (cDC1s) are characterized by XCR1 and CLEC9a markers in mice and BDCA-3 in humans [42]; and cDCs type 2 (cDC2s) are characterized by CD11b and SIRPα markers in mice and BDCA-1 in humans. The next subpopulation of dendritic cells is the monocyte group (moDCs), and they differentiate from monocytes exclusively under inflammatory conditions. This subpopulation of dendritic cells are also called “inflammatory” dendritic cells [43,44]. The last major subpopulation is represented by plasmacytoid dendritic cells (pDCs). They are characterized by Siglec-H markers in mice and BDCA-2 and CD123 in humans. cDC1s are capable of inducing antitumor immune responses through cross-presentation of tumor antigens to CD8+ T cells [45,46]. cDC1s are capable of engulfing necrotic or apoptotic tumor cells and presenting antigens to effector cells in the MHC class I complex, inducing the emergence of a population of antigen-specific CD8+ T cells [45,47]. The development of cDC1s depends on various transcription factors, including BATF3 [45] and IRF8 [48,49]. Genetic deletion of Batf 3 or point mutations in the Irf8 gene lead to the development of a cDC1 defect. With Batf3 deficiency, there is an increased frequency of development of cancer processors caused by a defect in tumor-specific CD8+ T-cell immunity [22,45]. Priming of naive CD8+ T cells has been shown to depend on the ability of cDC1s to migrate from tumors to regional lymph nodes [37] and cDC1s in the tumor microenvironment to support the cytotoxic activity of intratumoral CD8+ T cells [50]. The antitumor role of cDC1s is also realized through the active production of the cytokines CXCL9/CXCL10 and IL-12, which leads to activation and stimulation of infiltration of CD8+ T cells in tumors [51]. cDC1s are thought to be specialized not only for priming CD8+ T cells, but also for priming CD4+ T cells, which has a synergistic effect in mediating the antitumor cytotoxic immune response [52]. cDC2s efficiently present MHC class II antigens to trigger CD4+ T cells. They stimulate antitumor responses of CD4+ T cells and indirectly support the function of CD8+ T cells in the tumor microenvironment [41,53]. The presence of CD5+ cDC2s in tumors and regional lymph nodes is known to be associated with longer overall survival and disease-free survival of patients, as well as with immune rejection of tumors in mouse models [44,54,55]. CD5 on DCs potentiates the priming and activation of CD4+ T cells and CD8+ T cells. The role of inflammatory DC monocytes in antitumor immunity is well studied [56,57] and largely overlaps with the functions of cDC1s and cDC2s. They have been shown to stimulate CD4+ T cells in cancer [52] and to present opsonized tumor antigens and directly trigger CD8+ T cell responses [58,59].

The role of pDCs in antitumor immunity remains controversial. They weakly capture and present exogenous antigens but have the unique ability to produce huge amounts of IFNα in response to viral infection [60]. In patients with colon cancer, higher tumor infiltration of pDCs is associated with long-term survival [61], while in some cancer types, the presence of pDCs is associated with tumor progression and poor patient survival [62]. The effect of pDCs on antitumor immunity has not yet been fully studied. In addition, two new DC subtypes have recently been identified:

(1) The DC3 subtype, which was found in human peripheral blood, expresses the cDC2 marker (CD1c) and the monocyte marker (CD14) and has a different source of development to monocytes and common dendritic cell progenitors (CDPs), being a separate additional lineage of DCs [40,44,63].
(2) The transitional DC subtype (tDC) [64], which expresses Axl and is located on the border between cDC2 and pDC. The existence and function of these two DC subtypes in tumors remain to be determined (Table 1).

In conclusion, cDCs play a critical role in initiating and maintaining antitumor T-cell immunity [67,68], and the detection of cDCs in the tumor microenvironment is a favorable feature of cancer immunotherapy [69].

**Figure 1:**
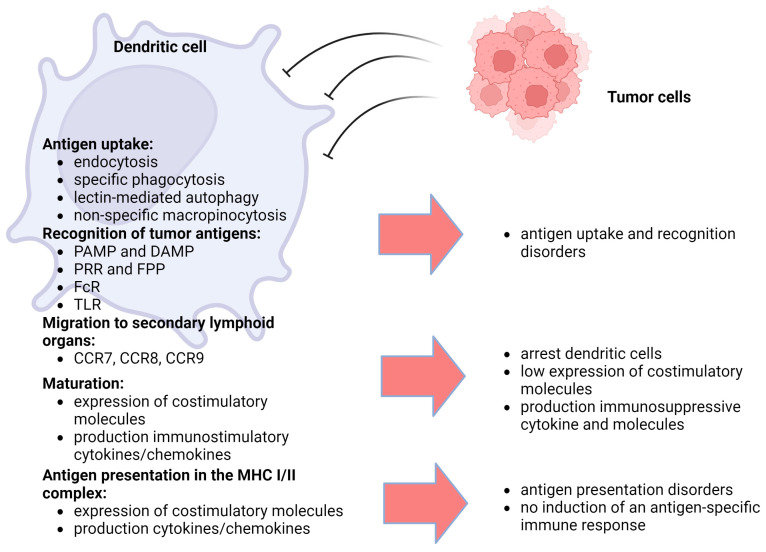
The main stages of antigen presentation by dendritic cells and their disorders. The main function of dendritic cells in the body is the capture, processing and presentation of antigens to immunocompetent cells; these processes involve a number of stages. Tumor cells have a negative impact on each of the stages of antigen presentation, suppressing the development of an antigen-specific immune response against them. This, on the one hand, leads to weak endocytosis of tumor cell components and processing inside dendritic cells. On the other hand, the maturation of dendritic cells is impaired as a result of inhibitors of immune cell differentiation secreted by tumor cells. As a result, dendritic cells remain immature, do not migrate to the lymph nodes, and have weak immunostimulating and antigen-presenting activity [92,93,94,95].

According to their ontogeny, dendritic cells can be divided into myeloid (conventional) or plasmacytoid dendritic cells. Furthermore, depending on their stage of development, dendritic cells can be divided into two main categories: immature and mature dendritic cells [70]. Most immature dendritic cells are located on the surface of mucous membranes, with skin and internal organs acting as sentinels for antigen recognition. These dendritic cells have lower expression of MHC I and MHC II, T cell costimulatory factors, and adhesion molecules [71]. Immature dendritic cells do not secrete proinflammatory cytokines, but they are capable of migration. When encountering an antigen, dendritic cells capture it and migrate to secondary lymphoid organs and present antigens to helper T cells or effector T cells to elicit specific cytotoxic T lymphocyte responses [72]. At the same time, they have increased expression of chemokine receptors (CCR7, -8 and -9) that determine their directed migration [73]. In summary, mature dendritic cells have a decreased ability to capture and process antigens but have an increased migratory capacity and increased expression of various costimulatory molecules (CD40, CD80 and CD86) and production of pro-inflammatory cytokines and chemokines [65]. Dendritic cells are very active, using their specific receptors to recognize foreign antigens or aberrant intrinsic antigens. Dendritic cells recognize antigens using molecular patterns: pathogen-associated molecular patterns (PAMPs), distress-associated molecular patterns (DAMPs) and pattern-recognition receptors (PRRs). Antigens are taken up by specific phagocytosis and nonspecific macropinocytosis. Phagocytosis is believed to be the defining mechanism of antigen uptake by dendritic cells and other immune cells [65]. There are two important forms of phagocytosis: microautophagy and lectin-mediated autophagy. The key endocytic protein involved in the process of microautophagy is the regulatory protein RAB5A, which has multiple effects (including an influence on cell membrane mobility and stiffness) [74]. C-type lectins and Fc receptors can recognize antigens by targeting specific ligands of apoptotic cells or pathogens, inducing the process of endocytosis [75]. One of the lectin receptors expressed on macrophages and dendritic cells is DC-SIGN (CD209), which is responsible for the binding of pathogens, which leads to their internalization into endosomes, where the pathogens are destroyed and an immune response is initiated [76,77]. It has been shown that high expression of annexin A2 in cancer was able to activate DC-SIGN and inhibit the dendritic cell-mediated antitumor immune response, which was accompanied by the suppression of dendritic cell maturation and IL-12 production and increased IL-10 production [78]. The role of formyl peptide receptors (FPRs) and Fc receptors (FcRs) expressed in hematopoietic cells and especially on dendritic cells should also be noted [79,80]. FPRs induce the migration of dendritic cells into the area of necrosis and apoptosis of tumor cells and influence tumor angiogenesis [81]. FcRs bind to various immunoglobulins (IgA, IgM, IgE and IgG) participating in antibody-mediated innate and adaptive immune responses, leading to the activation of CD4+ T cells [62,80,82]. A special role in the maturation and regulation of dendritic cell activity is played by toll-like receptors (TLRs) [83]. They are located on the surface of immune cells or intracellularly and recognize PAMPs for immune responses against pathogens and neoplastic cells. TLRs induce dendritic cell maturation (increased expression of CCR7, MHC-II, and co-stimulatory molecules CD80 or CD86) through activation of NF-kB [84,85]. TLRs are also expressed in tumor cells to evade the immune response [86]. Stimulation of TLR3 and TLR5 signal transduction can induce antitumor T-cell response; however, it was found that the risk of tumor process development increased against the background of chronic inflammation mediated by TLR4, TLR7, TLR8 and TLR9 [87]. Presentation of the captured antigen takes place directly in complex with MHC. MHC molecules are divided into two classes: MHC I and MHC II [88]. Both show a huge allelic polymorphism of the groove-binding peptide, which allows them to bind to a variety of peptides. MHC class I molecules are heterodimers consisting of two polypeptide chains: α- and β2-microglobulin [89]. MHC-II dimers have been shown to form in the endoplasmic reticulum and then bind to the non-polymorphic invariant chain Ii (CD 74). Peptide–MHC-II complexes are formed throughout the endocytic pathway, with antigen processing typically occurring in late endosomal compartments or lysosomes [90,91]. The efficiency and the process of antigen presentation itself are determined by a number of signals. Several signals are required to activate CD8+ or CD4+ T cells: signal 1, which is responsible for the binding of antigenic peptides to MHC-I or MHC-II molecules that are presented to CD8+ T cells or CD4+ T cells, respectively [92]; signal 2, whereby proper costimulatory signaling is ensured by a balance between a variety of positive (CD80/CD86) and negative signals (programmed death ligand 1 or 2 (PDL1/2)) on the dendritic cell surface [93,94]; and signal 3, whereby cytokines that stimulate T cells are produced by dendritic cells (IFN-γ and IL-12) [95]. These cytokines stimulate the functional expansion and development of memory CTL cells. Disruption of individual signals or their combinations leads to the disruption of natural mechanisms of antitumor defense (Figure 1).

Several antigen presentation pathways have been described, the main, classical one being antigen presentation to T cells via the T-cell receptor (TCR) [96]. MHC-I–peptide and MHC-II–peptide complexes on the surface of dendritic cells are presented to TCR complexes on CD8+ and CD4+ T cells, respectively. This triggers the processes of their activation of proliferation and differentiation [97]. Another pathway is represented by cross-presentation, a process by which dendritic cells capture, process and present extracellular antigens via MHC-I molecules to CD8+ T cells [98]. Cross-presentation is essential for CD8+ T-cell activation and has a significant impact on immune surveillance in transplantation and immune defense in infections. Only dendritic cells can cross-trigger the cytotoxic response of CD8+ T cells [99]. The described disorders of various links or mechanisms of tumor antigen presentation by dendritic cells are targets for correction and the development of new approaches and methods of cellular immunotherapy for cancer patients.

## 4. Cellular Immunotherapy Approaches Based on Antigen-Primed Dendritic Cells for Induction of Antitumor Immune Response

The existing approaches of cellular immunotherapy are based on obtaining functionally mature dendritic cells capable of presenting tumor antigens to effector cells and activating cytotoxic immune responses [100]. Given the marked suppression of immunity in cancer patients, and in order to eliminate the influence of tumors, the optimal option for the induction of functionally mature dendritic cells is to obtain them under in vitro conditions. Most clinical trials are based on dendritic cells derived ex vivo from peripheral blood monocytes. Typically, IL-4 and GM-CSF are used to induce dendritic cell differentiation from progenitor cells in 5–7 days [101,102] or 2–3 days, as in the case of “fast” dendritic cells [101,103]. Protocols for obtaining dendritic cells by culturing peripheral blood mononuclear cells (PBMCs) using various cytokines and cell differentiation factors (for example, IFN-β and IL-3 or GM-CSF) have been described [104,105]. Immature dendritic cells produce low levels of stimulatory cytokines and express fewer co-stimulatory molecules (CD80/CD86) [106,107].

The maturation and functional state of dendritic cells is assessed by the expression of classical dendritic cell markers, such as CD80, CD86, CD83 and CCR7 positivity, which can be measured using flow cytometry, but this is of limited use, since these markers are expressed by DCs with different phenotypes [108].

Experimental studies have shown that the use of dendritic cell maturation factors and cytokines that stimulate the development of the immune response (TLR agonists, CD40L and other cytokines (IL-12 and IL18)) induces a cytotoxic immune response in vitro [109,110].

**Figure 2:**
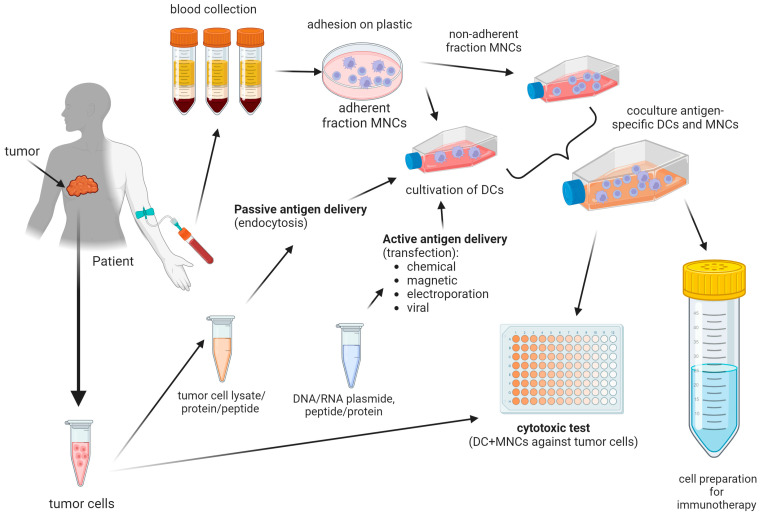
Schematic diagram of obtaining a cell preparation based on various forms and methods of delivering tumor antigens to dendritic cells. The production of modern cell preparations from dendritic cells is based on obtaining dendritic cells from patient peripheral blood monocytes under in vitro conditions. Various forms of delivery of tumor antigens are used: lysates of autologous tumor cells and isolated tumor-associated peptides/proteins or synthesized DNA or RNA constructs and peptides/proteins. These tumor antigens are delivered to dendritic cells passively, through natural endocytosis at the immature dendritic cell stage, or actively, through magnetic, chemical (lipofection), viral (transduction), transfection or electroporation methods. Currently, the greatest effectiveness in inducing an antitumor immune response is observed when co-cultivating in vitro obtained antigen-specific dendritic cells with lymphocytes (non-adhesive MNC fractions) to induce cytotoxic T-lymphocytes, including antigen-specific ones. The effectiveness of the resulting cell preparation is assessed in an in vitro cytotoxicity test against autologous tumor cells, if any, or cells of a tumor line expressing the target tumor-associated antigen [115,116,117].

Historically, approaches using in vitro induction of antigen-specific dendritic cells were initially used as monocellular therapies. The induction of antigen-specific dendritic cells alone in cell therapy showed limited efficacy. The best-known single-cell-type dendritic cell vaccine is the Provenge vaccine, which consists of autologous antigen-presenting cells (CD54+ cells) loaded with a recombinant fusion protein antigen that consists of GM-CSF and prostatic acid phosphatase. Clinical trials showed an increase in median survival of 4 months in patients with metastatic prostate cancer [111,112]. In subsequent works, scientists realized that a full-fledged antitumor cytotoxic response was produced by the activation of cytotoxic lymphocytes and the formation of a pool of antigen-specific effector cells. This is how the idea of optimal cell therapy as a polycellular therapy was formed, including the use of antigen-specific dendritic cells and antigen-primed CD8+ lymphocytes. The creation of such a cellular vaccine is a multistep process carried out in vitro (Figure 2). Accordingly, researchers have developed approaches to obtain dendritic cells capable of expressing tumor-associated antigens using various methods [110,113,114].

It should also be noted that the use of dendritic cell-based cellular immunotherapy has its own problems and limitations, including limited responses, possibly due to inefficient migration of dendritic cells from the injection site to the draining lymphoid organs and ineffective antigen presentation. As a result of the immunosuppressive effects of tumors, T cells activated by vaccination may be inactivated at the site of tumor growth. To prevent this, large numbers of dendritic cells and/or multiple doses of vaccine are required to ensure the production of sufficient numbers of activated cytotoxic T lymphocytes to kill tumor cells and ensure the production of sufficient numbers of memory T cells that will act as a rapid response in the event of tumor recurrence [108,115]. The number of cells required for immunotherapy requires their standardized production on a scale large enough to withstand repeated vaccinations. The production of cell-based vaccines requires highly specialized equipment and personnel to comply with current Good Manufacturing Practices (GMPs). The creation of cellular vaccines based on dendritic cells includes a number of key steps: collecting the patient’s peripheral blood and isolating mononuclear cells from it, collecting a tumor sample from the patient and isolating tumor cells and their antigens (cell lysates, RNA and DNA), obtaining mature dendritic cells loaded with tumor cells antigens (lysates, peptides, RNA and DNA), and obtaining a cellular vaccine based on dendritic cells.

The effectiveness of dendritic cell therapy may be limited by several factors, including failure to stimulate specific cytotoxic T lymphocytes, failure to activate NK cells or γδ T cells, failure to overcome the immunosuppressive effects of T regulatory cells and myeloid-derived suppressor cells (MDSCs), unwanted immune effects of T-regulatory cells and MDSCs, immunosuppression caused by decreased expression of tumor-associated antigens on tumor cells, overexpression of checkpoint proteins (checkpoint molecules), insufficient avidity of antigen-specific T lymphocytes, and a lack of suitable adjuvants that are required to induce TLR-mediated dendritic cell maturation.

1. Proteins or peptides and tumor cell lysates

One of the earliest methods used to deliver tumor-associated antigens is the use of lysates and tumor proteins (peptides) from autologous tumor cells to prime dendritic cells [118,119], which express a wider range of tumor antigens suitable for personalized treatment, and tumor cells fused with dendritic cell vaccines [119,120]. An alternative method for creating a vaccine based on dendritic cells is the production of whole-tumor vaccines, which use whole or lysed tumor cells, both intact and modified, as a source of antigens and other immunogenic factors to stimulate the antitumor immune response. This approach, as with the use of tumor cell lysates, provides a complete set of tumor antigens [121]. In the case of dendritic cells, autologous or allogeneic tumor cells are used, pretreated to impart increased immunogenicity and increase therapeutic efficacy [122,123].

The use of tumor lysates as a source of tumor immunogens has the potential advantage of stimulating responses against multiple individual tumor-associated antigens. This approach allows for the induction of a polyclonal immune response by stimulating both CD4+ T cells and CD8+ T cells. The use of tumor lysates reduces the time and effort required to identify and synthesize individual immunodominant peptide epitopes, allowing dendritic cells to naturally process tumor antigens. The disadvantage of this method is the impossibility, in a number of clinical situations, of obtaining tumor material for preparing a lysate [124]. The effectiveness of the vaccine depends on the concentration of immunogenic and immunosuppressive antigens in the tumor material [125]. In addition, the use of tumor cell lysate antigens does not capture the changing repertoire of tumor antigens that occurs during metastasis, as well as during specific chemotherapy and/or radiation therapy. With this activation method, there is a potential risk of developing autoimmune reactions, since normal tissue cells may be present in the tumor material. However, a tumor antigen presented by dendritic cells has been shown to stimulate a specific antitumor cytotoxic response [126].

Thus, it was shown in cellular immunotherapy of breast cancer patients that the administration of dendritic cells primed with lysates of autologous breast cancer tumor cells led to an increase in the percentage of patients with a relapse-free course, which was accompanied by a decrease in the content of suppressor cells (Treg and CD14+ HLA-DR) in the peripheral blood and an increase in the content of circulating myeloid dendritic cells in the peripheral blood, compared to before immunotherapy [127].

The use of a full-length tumor protein as a tumor antigen has a number of positive aspects. In particular, the selection of a tumor protein by HLA haplotyping becomes irrelevant, which avoids the question of the need to identify individual epitopes. This method has been successfully used in clinical studies of cell-based vaccines against lung cancer, kidney cancer, lymphoma and myeloma, showing the formation of an antigen-specific cytotoxic response [128]. However, it is worth noting that when dendritic cells are loaded with an extracellular antigen, effective presentation of its epitopes in combination with MHC class I and stimulation of CD8+ T cells does not occur. Therefore, at present, this approach is only used in combination with other approaches to increase the immunogenicity of a protein. For example, hybrid proteins may contain the TAT protein of the human immunodeficiency virus, which improves penetration into the cell [129,130].

Modern technologies make it possible to create and use synthetic peptides to load antigens into MHC class I or II complexes, depending on the epitope, and induce an epitope-specific T-cell response [131]. This method also reduces the risk of autoimmune reactions because only the epitope of the tumor protein is used and there is no cross-reaction with the patient’s own tissue. The biggest disadvantage of this method is the mandatory determination of antigen epitopes, the human HLA type and the amino acid sequence in a given peptide. The use of a peptide does not allow one to accurately predict the processing of these molecules, and, as a result, it is not always possible to present the necessary antigens in their native form. In addition, the use of peptides as a source of antigens is limited by the size of the molecules that can enter the cell or bind to MHC on the surface of antigen-presenting cells [132]. Currently, this approach is aimed primarily at the CD8+-mediated cytotoxic antitumor immune response, though, to a lesser extent, it also stimulates the CD4+ T-cell response [133,134].

Thus, when using dendritic cells loaded with a peptide/lysate, a number of difficulties arise in controlling the final cytotoxic effect. This is due to the fact that the duration of tumor antigen expression by dendritic cells is limited by the affinity of the peptide for the MHC molecule and the half-life of the peptide–MHC complex [135,136]. When producing cellular vaccines using tumor cells, a number of problems arise related to the standardization of tumor-associated antigens and the availability of tumor cells. In such cases, an alternative is to use tumor cell RNA for delivery to dendritic cells. To obtain RNA from tumor cells, a small number of cells are required, which can be obtained from a tumor biopsy. This method is also useful in situations where tumor-specific antigens cannot be detected on the surface of tumor cells.

2. DNA and RNA constructs

These vaccines are created using DNA or RNA fragments that encode epitopes of TAAs. In addition, these DNA and RNA fragments are delivered to dendritic cells in the form of synthesized plasmids by transfection (chemical, magnetic or electroporative).

The introduction of DNA/RNA constructs into dendritic cells has a number of advantages compared to cells loaded with peptides/lysates ex vivo and tumor cells [114]. Through software, it is possible to design a DNA construct that includes only the most immunogenic epitopes of a tumor-associated antigen, while autoimmune and immunosuppressive epitopes are excluded. The use of DNA constructs makes it possible to specifically modulate the immune response against tumor cells expressing a given antigen, while several genes (including those differing with respect to HLA haplotype specificity) encoding various antigens can be included in one DNA plasmid [137]. As a result of transfection of dendritic cells with DNA constructs, long-term expression of the tumor antigen occurs. When genetic material is introduced into dendritic cells, antigens undergo endogenous processing for presentation in complex with MHC class I molecules, which leads to effective stimulation of a cytotoxic antitumor immune response. After processing, the cells express the antigens in their native form, which facilitates their processing and presentation to the immune system. The use of polyepitope constructs makes it possible to trigger the reaction of several clones of T cells, ensuring the launch of a more powerful immune response against various cancer cells that are part of the tumor, and, in addition, it allows the possible loss of expression of a given tumor-associated antigen in tumor cells to be overcome. HLA typing allows the selection of only those epitopes that increase the likelihood of developing a specific CD8+ or CD4+ T-cell response [138].

In addition to using the sequences of specific immunogenic epitopes of tumor antigens, constructs can be added to the plasmid DNA/RNA that affect various stages of signal transduction between dendritic cells and T cells, including:

- Genetic modifications to ensure the delivery of antigens to stimulate T-cell receptors. This is achieved by using HLA-specific epitope sequences, enhancing endogenous antigen expression by dendritic cells and sufficient and continuous delivery of naturally processed antigens;
- Genetic modifications to enhance costimulatory signals. This is achieved either by enhancing costimulatory signals or by suppressing the expression of inhibitory molecules;
- Genetic modifications aimed at improving the immune microenvironment, for example, stimulating the secretion of Th1 cytokines (TNF-α, IFN-γ, IL-2 and IL-18), suppressing the activity of regulatory cytokines (TGF-β and IL-10) and changing the secretion of chemokines.

Transfection of DNA/RNA constructs encoding tumor antigens into dendritic cells is carried out by plasmid transfection methods (liposome-mediated transfection, magnetic transfection and other types of transfection), viral vectors or electroporation [139]. The main disadvantage of using genetic constructs is the low efficiency (about 5–20%) of their direct uptake by dendritic cells [140]. The delivery method used for gene transfer has been suggested to have a major impact on transfection efficiency [135]. The weak transfection efficiency may be due to the limited ability of DNA molecules to reach the nucleus, where transcription occurs [141]. In addition, physical methods can disrupt and alter the function and phenotype of dendritic cells, or even be toxic to the cells [142]. On the other hand, the use of electroporation has shown an increase in transfection efficiency and cell viability [142,143]. Although viral vectors are much more effective at penetrating dendritic cells (about 90–100%), their use in dendritic cell vaccine trials is limited due to possible viral damage to the cells.

Thus, to optimally select a source of tumor antigens for priming dendritic cells, the following factors must be taken into account:

1. Certain peptide/DNA/RNA molecules contain epitopes specific for a certain HLA type, and, accordingly, the effectiveness of their use will be limited to a certain human HLA type;
2. The presence of a limited set of well-characterized tumor-associated antigens and the risks of changes in the antigenic profiles of target tumor cells as a result of malformation and/or exposure to chemotherapy;
3. When using certain peptide/DNA/RNA molecules, the repertoire of T cell clones is limited and, accordingly, the ability of the immune system to produce a strong multispecific antitumor response is limited.

Dendritic cell vaccines have shown good results in preclinical trials [140]. The use of dendritic cells is sufficient to activate the T cell response, but they are not always able to provide adequate additional support for the immune response. It is known that a tumor is capable of creating an immunosuppressive microenvironment. Therefore, it is necessary to develop successful dendritic cell-based vaccines that provide a strong and durable immune response.

An alternative approach that is increasingly being used is to transfect DCs with RNA or DNA encoding tumor-associated antigens and their immunogenic epitopes, which through continuous gene expression can maintain antigen expression throughout the life of the dendritic cells [117].

Therefore, combination immunotherapies, such as immune checkpoint inhibitors (ICIs) and autologous antigen-loaded DC vaccination, are recommended to improve clinical outcomes and address the challenges associated with dendritic cell vaccination. Neoantigens are recommended as a source of antigens for the development of DC vaccines because they have lower immunological tolerance and are less toxic than tumor-associated antigens [144].

Thus, further development of dendritic cell-based immunotherapy technology aims to enhance and activate the immune response against cancer by harnessing the potent immunostimulatory properties of dendritic cells to maximize their ability to capture tumor antigens, stimulate T cells and trigger an antigen-specific antitumor immune response [145]. Vaccination with antigen-specific dendritic cells and adoptive transfer of cytotoxic cells primed with the resulting antigen-specific dendritic cells, in combination with combination therapy, form the basis of modern cancer immunotherapy. The ex vivo generation of antigen-specific cytotoxic T cells using antigen-primed dendritic cells is currently implemented using TCR T cell technology [146].

Therefore, it has become necessary to search for more specific antigenic substrates (immunogenic epitopes of tumor-associated antigens). Due to the development of technologies, it has become possible to obtain immunogenic epitopes of tumor-associated antigens and to create DNA and RNA constructs on their basis to obtain genetically modified dendritic cells [147,148]. This approach has made it possible to include in the composition of plasmids information not only about tumor-associated antigen epitopes but also about “checkpoint” molecules, which markedly increases the induction of cytotoxic immune responses. Delivery of genetic constructs (DNA and RNA) into dendritic cells is carried out by various methods (chemical or magnetic transfection, electroporation and viral transduction), the essence of which is the transportation of genetic information encoded in plasmids (DNA and RNA) inside the cell, bypassing the process of endocytosis and targeted plasmid processing.

In preclinical studies, the above delivery modes were shown to be significantly effective in the induction of antitumor immune responses. In in vivo (mouse) and clinical studies, different modes of delivery of cellular vaccines based on autologous dendritic cells were used. Thus, different methods have been used to deliver dendritic cell-based vaccines to patients, such as intravenous [149], intradermal [150], and, less frequently, intranodal [151] and intratumoral routes [152,153], as well as in vivo dendritic cell induction [154]. At the same time, antigen-loaded dendritic cells can stimulate T-cell immunity regardless of the route of administration [152].

Thus, an approach using DNA constructs encoding epitopes of tumor-associated antigens of various cancers (breast cancer, colorectal cancer and non-small cell lung cancer) for the induction of antigen-specific immune responses against autologous tumor cells in vitro was successfully demonstrated [109]. A number of authors used the approach of inducing dendritic cells by delivery of CD40L, CD70 and TLR4 mRNAs, resulting in the formation of mature dendritic cells in a one-step process without further incubation with other maturation cocktails. Synthetic mRNA-based vaccines caused fewer side effects and showed higher potential for optimization and large-scale production than dendritic cell vaccines based on whole-tumor mRNA [70,155,156]. The described approach stimulated antigen-specific CD8+ T-lymphocyte cells in vaccinated patients [157,158,159]. Genetic constructs also include various transcription factors (PU.1, Irf8 and Batf3) and migration factor genes (SSR7) to obtain mature dendritic cells with a notable potential for migration to draining lymph nodes [160,161]. It was shown that dendritic cells transfected with DNA constructs encoding Her2/neu epitopes induced the formation of not only a population of antigen-specific CD8+ T cells but also memory T cells [115,162,163,164]. In addition to approaches using genetic constructs to activate dendritic cell maturation and induce cytotoxic T lymphocytes, plasmids encoding receptors specific to certain tumor-associated antigens were used to selectively uptake extracellular vesicles of tumor origin that can deliver tumor-associated antigens to dendritic cells and/or encode small interfering RNAs to repress PD-L1 and PD-L2 genes in dendritic cells [165]. PD-1, when stimulated on the surface of T cells, induces antigen-specific anergy or apoptosis [161,166]. Many types of tumors, as well as mature dendritic cells, express PD ligands (PD-L1) on their cell surfaces [167]. Dendritic cells found in tumor-draining lymph nodes in an ovarian carcinoma model express high levels of PD-L1, and blockade of PD-L1 enhances the activation of cytotoxic T lymphocytes by dendritic cells and shifts cytokine production from a Th2 response to a Th1 response [168]. Blocking immunosuppressive signaling is one of the current approaches in cancer vaccines [169,170]. It should be noted that, in addition to approaches used to obtain functionally active dendritic cells by priming cytotoxic T-lymphocytes, which provide the necessary first two signals for activation of CD8+ or CD4+ T-cells (Figure 1), cytokines are also used to activate T-lymphocytes (IL-12, IL-18 and IFN-γ) [109] and generate antigen-specific cytotoxic T-lymphocytes (IL-2, IL-7 and IL-15) [163].

Cell therapy is personalized, and courses of therapy and cell preparations are created individually for each patient. This requires specialized laboratory equipment and skills to isolate a subpopulation of dendritic cells from the patient’s blood or cultivate them in vitro from progenitor cells, delivering antigens using high-tech transfection and targeted delivery methods [126].

Along with this, it should be noted that the use of cell therapy based on dendritic cells is safe; the local and systemic reactions (rash, itching and fever) that have been described are rare, in contrast to those that have been described for other immunotherapy approaches, such as graft-versus-host disease (GVHD) and immunosuppression (with allogeneic bone marrow transplantation), cytokine storm syndrome, neurotoxicity (with CAR T-cell therapy), and autoimmune reactions (with blockade of CTLA-4 or PD-1) [171,172].

Thus, the existing technologies make it possible to perform in vitro the whole chain of induction of antigen-specific cytotoxic immune responses. Genetically engineered dendritic cell vaccines expressing tumor-associated antigens have shown significant efficacy against many types of cancer [173].

## 5. Conclusions

The development of cell technologies based on dendritic cells evolved as dendritic cell differentiation disorders and their functions in cancer, the mechanisms of tumor antigen presentation and priming of cytotoxic T-lymphocytes, and the induction of antigen-specific antitumor immune responses were studied. At the initial stage of technology development, protocols for in vitro generation of functionally mature dendritic cells capable of capturing tumor antigens and processing and presenting them in complex with MHC to T-lymphocytes were developed. Based on this, various forms of tumor-associated antigen delivery (lysates or proteins (peptides) from tumor cells and DNA and RNA constructs) were tested, and it was shown that the use of DNA and RNA constructs allowed not only for the delivery of the most immunogenic epitopes of tumor-associated antigens to dendritic cells but also for the enhancement of their ability to induce antigen-specific cytotoxic T-lymphocytes. Currently, cell therapy based on dendritic cells, due to the possibility of creating DNA and RNA constructs encoding information about both target tumor antigens and regulatory molecules, is a modern basis for antigen-specific immunotherapy of cancer. In the future, the development of antigen-specific cell therapy will be aimed at studying the functional activity of antigen-specific cytotoxic T-lymphocytes induced by dendritic cells and developing approaches to obtain them in vitro in the necessary quantities for therapy.

## Figures and Tables

**Table 1:** Biomarkers of various blood dendritic cell subtypes and their biological functions.

Dendritic Cell Subset	Biomarkers	Secretory Cytokines	Biological Functions	References
Conventional (myeloid) DCs type 1 (cDC1s)	CD11c MHC II BDCA-3 (CD141) XCR1 CLEC9A IRF8	IL-12 TNF-α IL-6	Inducing antitumor immune response through cross-presentation of tumor antigens to CD8+ T cells	[42,45,46,47,51,52,65]
Conventional (myeloid) DCs type 2 (cDC2s)	CD11c MHC II BDCA-1 (CD1c) CD172a CD11b IRF4 CLEC10A	IL-1β IL-6 IL-12 IL-23 IL-10 TNF-α TGF-β	Presentation of MHC class II antigens, triggering CD4+ T cells	[41,53]
“Inflammatory” DCs (moDCs)	CD11c MHC II CD11b CD14 CD209 CD226 CD1a/b/c CD206 IRF4 CD172a CD64 CD88 CLEC10A	IL-1β IL-6 IL-10 IL-12 IL-23 TNF-α	Direct presentation of antigens to CD4+ T cells, induction of Th1 and Th17 responses, cross-presentation capability	[43,44,56,57]
Plasmacytoid DCs (pDCs)	MHC II BDCA-2 (CD303) BDCA-4 (CD304) CD123 CD45RA	IFN-α TNF-α	Induction of antiviral immune response	[60]
DCs type 3 (DC3s)	CD1c+ CD14+ MHC II BDCA-3 (CD141) BDCA-1 (CD1c) CD163 CLEC10A	IL-1β IL-6 IL-23 TNF-α IL-10	Presentation of MHC class II antigens, triggering CD4+ T cells	[40,44,63,66]
Transitional DC subtype (tDCs)	CD11c^low/high^ AXL+ SIGLEC6+	IL-1β	Presentation of MHC class II antigens, triggering CD4+ T cells	[64]

## References

[1] Xin H.-W., Ambe C.M., Hari D.M., Wiegand G.W., Miller T.C., Chen J.-Q., Anderson A.J., Ray S., Mullinax J.E., Koizumi T. (2013). Label-retaining liver cancer cells are relatively resistant to sorafenib. Gut.

[2] Xin H.-W., Hari D.M., Mullinax J.E., Ambe C.M., Koizumi T., Ray S., Anderson A.J., Wiegand G.W., Garfield S.H., Thorgeirsson S.S. (2012). Tumor-initiating label-retaining cancer cells in human gastrointestinal cancers undergo asymmetric cell division. Stem Cells.

[3] Liu Y., Yu C., Wu Y., Sun X., Su Q., You C., Xin H. (2017). CD44+ fibroblasts increases breast cancer cell survival and drug resistance via IGF2BP3-CD44-IGF2 signalling. J. Cell. Mol. Med..

[4] Paek S.H., Kim H.G., Lee J.W., Woo J., Kwon H., Kim J.B., Lim W., Kim J.R., Moon B.-I., Paik N.-S. (2019). Circulating Plasmacytoid and Myeloid Dendritic Cells in Breast Cancer Patients: A Pilot Study. J. Breast Cancer.

[5] Chevolet I., Speeckaert R., Schreuer M., Neyns B., Krysko O., Bachert C., van Gele M., van Geel N., Brochez L. (2015). Clinical significance of plasmacytoid dendritic cells and myeloid-derived suppressor cells in melanoma. J. Transl. Med..

[6] Hirooka S., Yanagimoto H., Satoi S., Yamamoto T., Toyokawa H., Yamaki S., Yui R., Inoue K., Michiura T., Kwon A.-H. (2011). The role of circulating dendritic cells in patients with unresectable pancreatic cancer. Anticancer Res..

[7] Gabrilovich D.I., Ostrand-Rosenberg S., Bronte V. (2012). Coordinated regulation of myeloid cells by tumours. Nat. Rev. Immunol..

[8] Gabrilovich D. (2004). Mechanisms and functional significance of tumour-induced dendritic-cell defects. Nat. Rev. Immunol..

[9] Lee B.-N., Follen M., Rodriquez G., Shen D.-Y., Malpica A., Shearer W.T., Reuben J.M. (2006). Deficiencies in myeloid antigen-presenting cells in women with cervical squamous intraepithelial lesions. Cancer.

[10] Ormandy L.-A., Farber A., Cantz T., Petrykowska S., Wedemeyer H., Horning M., Lehner F., Manns M.-P., Korangy F., Greten T.-F. (2006). Direct ex vivo analysis of dendritic cells in patients with hepatocellular carcinoma. World J. Gastroenterol..

[11] Perrot I., Blanchard D., Freymond N., Isaac S., Guibert B., Pachéco Y., Lebecque S. (2007). Dendritic cells infiltrating human non-small cell lung cancer are blocked at immature stage. J. Immunol..

[12] Chaux P., Moutet M., Faivre J., Martin F., Martin M. (1996). Inflammatory cells infiltrating human colorectal carcinomas express HLA class II but not B7-1 and B7-2 costimulatory molecules of the T-cell activation. Lab. Investig. A J. Tech. Methods Pathol..

[13] Pinzon-Charry A., Ho C.S.K., Maxwell T., McGuckin M.A., Schmidt C., Furnival C., Pyke C.M., López J.A. (2007). Numerical and functional defects of blood dendritic cells in early- and late-stage breast cancer. Br. J. Cancer.

[14] Gabrilovich D.I., Chen H.L., Girgis K.R., Cunningham H.T., Meny G.M., Nadaf S., Kavanaugh D., Carbone D.P. (1996). Production of vascular endothelial growth factor by human tumors inhibits the functional maturation of dendritic cells. Nat. Med..

[15] Gabrilovich D., Ishida T., Oyama T., Ran S., Kravtsov V., Nadaf S., Carbone D.P. (1998). Vascular endothelial growth factor inhibits the development of dendritic cells and dramatically affects the differentiation of multiple hematopoietic lineages in vivo. Blood.

[16] Lissoni P., Malugani F., Bonfanti A., Bucovec R., Secondino S., Brivio F., Ferrari-Bravo A., Ferrante R., Vigoré L., Rovelli F. (2001). Abnormally enhanced blood concentrations of vascular endothelial growth factor (VEGF) in metastatic cancer patients and their relation to circulating dendritic cells, IL-12 and endothelin-1. J. Biol. Regul. Homeost. Agents.

[17] Wherry E.J. (2011). T cell exhaustion. Nat. Immunol..

[18] Crespo J., Sun H., Welling T.H., Tian Z., Zou W. (2013). T cell anergy, exhaustion, senescence, and stemness in the tumor microenvironment. Curr. Opin. Immunol..

[19] Brahmer J.R., Tykodi S.S., Chow L.Q.M., Hwu W.-J., Topalian S.L., Hwu P., Drake C.G., Camacho L.H., Kauh J., Odunsi K. (2012). Safety and activity of anti-PD-L1 antibody in patients with advanced cancer. N. Engl. J. Med..

[20] Topalian S.L., Hodi F.S., Brahmer J.R., Gettinger S.N., Smith D.C., McDermott D.F., Powderly J.D., Carvajal R.D., Sosman J.A., Atkins M.B. (2012). Safety, activity, and immune correlates of anti-PD-1 antibody in cancer. N. Engl. J. Med..

[21] Jochems C., Schlom J. (2011). Tumor-infiltrating immune cells and prognosis: The potential link between conventional cancer therapy and immunity. Exp. Biol. Med..

[22] Salmon H., Idoyaga J., Rahman A., Leboeuf M., Remark R., Jordan S., Casanova-Acebes M., Khudoynazarova M., Agudo J., Tung N. (2016). Expansion and Activation of CD103^+^ Dendritic Cell Progenitors at the Tumor Site Enhances Tumor Responses to Therapeutic PD-L1 and BRAF Inhibition. Immunity.

[23] Maurya N., Gujar R., Gupta M., Yadav V., Verma S., Sen P. (2014). Immunoregulation of dendritic cells by the receptor T cell Ig and mucin protein-3 via Bruton’s tyrosine kinase and c-Src. J. Immunol..

[24] de Mingo Pulido Á., Gardner A., Hiebler S., Soliman H., Rugo H.S., Krummel M.F., Coussens L.M., Ruffell B. (2018). TIM-3 Regulates CD103+ Dendritic Cell Function and Response to Chemotherapy in Breast Cancer. Cancer Cell.

[25] Michielsen A.J., Hogan A.E., Marry J., Tosetto M., Cox F., Hyland J.M., Sheahan K.D., O’Donoghue D.P., Mulcahy H.E., Ryan E.J. (2011). Tumour tissue microenvironment can inhibit dendritic cell maturation in colorectal cancer. PLoS ONE.

[26] Spranger S., Bao R., Gajewski T.F. (2015). Melanoma-intrinsic β-catenin signalling prevents anti-tumour immunity. Nature.

[27] Zelenay S., van der Veen A.G., Böttcher J.P., Snelgrove K.J., Rogers N., Acton S.E., Chakravarty P., Girotti M.R., Marais R., Quezada S.A. (2015). Cyclooxygenase-Dependent Tumor Growth through Evasion of Immunity. Cell.

[28] Gottfried E., Kunz-Schughart L.A., Ebner S., Mueller-Klieser W., Hoves S., Andreesen R., Mackensen A., Kreutz M. (2006). Tumor-derived lactic acid modulates dendritic cell activation and antigen expression. Blood.

[29] Tyurin V.A., Cao W., Tyurina Y.Y., Gabrilovich D.I., Kagan V.E. (2011). Mass-spectrometric characterization of peroxidized and hydrolyzed lipids in plasma and dendritic cells of tumor-bearing animals. Biochem. Biophys. Res. Commun..

[30] Cubillos-Ruiz J.R., Silberman P.C., Rutkowski M.R., Chopra S., Perales-Puchalt A., Song M., Zhang S., Bettigole S.E., Gupta D., Holcomb K. (2015). ER Stress Sensor XBP1 Controls Anti-tumor Immunity by Disrupting Dendritic Cell Homeostasis. Cell.

[31] Ramakrishnan R., Tyurin V.A., Veglia F., Condamine T., Amoscato A., Mohammadyani D., Johnson J.J., Zhang L.M., Klein-Seetharaman J., Celis E. (2014). Oxidized lipids block antigen cross-presentation by dendritic cells in cancer. J. Immunol..

[32] Veglia F., Tyurin V.A., Mohammadyani D., Blasi M., Duperret E.K., Donthireddy L., Hashimoto A., Kapralov A., Amoscato A., Angelini R. (2017). Lipid bodies containing oxidatively truncated lipids block antigen cross-presentation by dendritic cells in cancer. Nat. Commun..

[33] Ito T., Yang M., Wang Y.-H., Lande R., Gregorio J., Perng O.A., Qin X.-F., Liu Y.-J., Gilliet M. (2007). Plasmacytoid dendritic cells prime IL-10-producing T regulatory cells by inducible costimulator ligand. J. Exp. Med..

[34] Aspord C., Leccia M.-T., Charles J., Plumas J. (2013). Plasmacytoid dendritic cells support melanoma progression by promoting Th2 and regulatory immunity through OX40L and ICOSL. Cancer Immunol. Res..

[35] Lombardi V.C., Khaiboullina S.F., Rizvanov A.A. (2015). Plasmacytoid dendritic cells, a role in neoplastic prevention and progression. Eur. J. Clin. Investig..

[36] Saadeh D., Kurban M., Abbas O. (2016). Plasmacytoid dendritic cell role in cutaneous malignancies. J. Dermatol. Sci..

[37] Roberts E.W., Broz M.L., Binnewies M., Headley M.B., Nelson A.E., Wolf D.M., Kaisho T., Bogunovic D., Bhardwaj N., Krummel M.F. (2016). Critical Role for CD103^+^/CD141^+^ Dendritic Cells Bearing CCR7 for Tumor Antigen Trafficking and Priming of T Cell Immunity in Melanoma. Cancer Cell.

[38] Anguille S., Smits E.L., Bryant C., van Acker H.H., Goossens H., Lion E., Fromm P.D., Hart D.N., van Tendeloo V.F., Berneman Z.N. (2015). Dendritic Cells as Pharmacological Tools for Cancer Immunotherapy. Pharmacol. Rev..

[39] Fang P., Li X., Dai J., Cole L., Camacho J.A., Zhang Y., Ji Y., Wang J., Yang X.-F., Wang H. (2018). Immune cell subset differentiation and tissue inflammation. J. Hematol. Oncol..

[40] Villani A.-C., Satija R., Reynolds G., Sarkizova S., Shekhar K., Fletcher J., Griesbeck M., Butler A., Zheng S., Lazo S. (2017). Single-cell RNA-seq reveals new types of human blood dendritic cells, monocytes, and progenitors. Science.

[41] Brown C.C., Gudjonson H., Pritykin Y., Deep D., Lavallée V.-P., Mendoza A., Fromme R., Mazutis L., Ariyan C., Leslie C. (2019). Transcriptional Basis of Mouse and Human Dendritic Cell Heterogeneity. Cell.

[42] Bachem A., Güttler S., Hartung E., Ebstein F., Schaefer M., Tannert A., Salama A., Movassaghi K., Opitz C., Mages H.W. (2010). Superior antigen cross-presentation and XCR1 expression define human CD11c+CD141+ cells as homologues of mouse CD8+ dendritic cells. J. Exp. Med..

[43] Cheong C., Matos I., Choi J.-H., Dandamudi D.B., Shrestha E., Longhi M.P., Jeffrey K.L., Anthony R.M., Kluger C., Nchinda G. (2010). Microbial stimulation fully differentiates monocytes to DC-SIGN/CD209^+^ dendritic cells for immune T cell areas. Cell.

[44] Cytlak U., Resteu A., Pagan S., Green K., Milne P., Maisuria S., McDonald D., Hulme G., Filby A., Carpenter B. (2020). Differential IRF8 Transcription Factor Requirement Defines Two Pathways of Dendritic Cell Development in Humans. Immunity.

[45] Hildner K., Edelson B.T., Purtha W.E., Diamond M., Matsushita H., Kohyama M., Calderon B., Schraml B.U., Unanue E.R., Diamond M.S. (2008). Batf3 deficiency reveals a critical role for CD8alpha+ dendritic cells in cytotoxic T cell immunity. Science.

[46] Bedoui S., Whitney P.G., Waithman J., Eidsmo L., Wakim L., Caminschi I., Allan R.S., Wojtasiak M., Shortman K., Carbone F.R. (2009). Cross-presentation of viral and self antigens by skin-derived CD103+ dendritic cells. Nat. Immunol..

[47] Broz M.L., Binnewies M., Boldajipour B., Nelson A.E., Pollack J.L., Erle D.J., Barczak A., Rosenblum M.D., Daud A., Barber D.L. (2014). Dissecting the tumor myeloid compartment reveals rare activating antigen-presenting cells critical for T cell immunity. Cancer Cell.

[48] Zhang S., Audiger C., Chopin M., Nutt S.L. (2023). Transcriptional regulation of dendritic cell development and function. Front. Immunol..

[49] Hambleton S., Salem S., Bustamante J., Bigley V., Boisson-Dupuis S., Azevedo J., Fortin A., Haniffa M., Ceron-Gutierrez L., Bacon C.M. (2011). IRF8 mutations and human dendritic-cell immunodeficiency. N. Engl. J. Med..

[50] Spranger S., Dai D., Horton B., Gajewski T.F. (2017). Tumor-Residing Batf3 Dendritic Cells Are Required for Effector T Cell Trafficking and Adoptive T Cell Therapy. Cancer Cell.

[51] Garris C.S., Arlauckas S.P., Kohler R.H., Trefny M.P., Garren S., Piot C., Engblom C., Pfirschke C., Siwicki M., Gungabeesoon J. (2018). Successful Anti-PD-1 Cancer Immunotherapy Requires T Cell-Dendritic Cell Crosstalk Involving the Cytokines IFN-γ and IL-12. Immunity.

[52] Ferris S.T., Durai V., Wu R., Theisen D.J., Ward J.P., Bern M.D., Davidson J.T., Bagadia P., Liu T., Briseño C.G. (2020). cDC1 prime and are licensed by CD4+ T cells to induce anti-tumour immunity. Nature.

[53] Maier B., Leader A.M., Chen S.T., Tung N., Chang C., LeBerichel J., Chudnovskiy A., Maskey S., Walker L., Finnigan J.P. (2020). A conserved dendritic-cell regulatory program limits antitumour immunity. Nature.

[54] Yin X., Yu H., Jin X., Li J., Guo H., Shi Q., Yin Z., Xu Y., Wang X., Liu R. (2017). Human Blood CD1c+ Dendritic Cells Encompass CD5high and CD5low Subsets That Differ Significantly in Phenotype, Gene Expression, and Functions. J. Immunol..

[55] Korenfeld D., Gorvel L., Munk A., Man J., Schaffer A., Tung T., Mann C., Klechevsky E. (2017). A type of human skin dendritic cell marked by CD5 is associated with the development of inflammatory skin disease. JCI Insight.

[56] Sharma M.D., Rodriguez P.C., Koehn B.H., Baban B., Cui Y., Guo G., Shimoda M., Pacholczyk R., Shi H., Lee E.-J. (2018). Activation of p53 in Immature Myeloid Precursor Cells Controls Differentiation into Ly6c+CD103+ Monocytic Antigen-Presenting Cells in Tumors. Immunity.

[57] Schetters S.T.T., Rodriguez E., Kruijssen L.J.W., Crommentuijn M.H.W., Boon L., van den Bossche J., Den Haan J.M.M., van Kooyk Y. (2020). Monocyte-derived APCs are central to the response of PD1 checkpoint blockade and provide a therapeutic target for combination therapy. J. Immunother. Cancer.

[58] Bosteels C., Neyt K., Vanheerswynghels M., van Helden M.J., Sichien D., Debeuf N., de Prijck S., Bosteels V., Vandamme N., Martens L. (2020). Inflammatory Type 2 cDCs Acquire Features of cDC1s and Macrophages to Orchestrate Immunity to Respiratory Virus Infection. Immunity.

[59] Cohn L., Chatterjee B., Esselborn F., Smed-Sörensen A., Nakamura N., Chalouni C., Lee B.-C., Vandlen R., Keler T., Lauer P. (2013). Antigen delivery to early endosomes eliminates the superiority of human blood BDCA3+ dendritic cells at cross presentation. J. Exp. Med..

[60] Reizis B. (2019). Plasmacytoid Dendritic Cells: Development, Regulation, and Function. Immunity.

[61] Kießler M., Plesca I., Sommer U., Wehner R., Wilczkowski F., Müller L., Tunger A., Lai X., Rentsch A., Peuker K. (2021). Tumor-infiltrating plasmacytoid dendritic cells are associated with survival in human colon cancer. J. Immunother. Cancer.

[62] Cervenak J., Kurrle R., Kacskovics I. (2015). Accelerating antibody discovery using transgenic animals overexpressing the neonatal Fc receptor as a result of augmented humoral immunity. Immunol. Rev..

[63] Villar J., Segura E. (2020). The More, the Merrier: DC3s Join the Human Dendritic Cell Family. Immunity.

[64] Sulczewski F.B., Maqueda-Alfaro R.A., Alcántara-Hernández M., Perez O.A., Saravanan S., Yun T.J., Seong D., Arroyo Hornero R., Raquer-McKay H.M., Esteva E. (2023). Transitional dendritic cells are distinct from conventional DC2 precursors and mediate proinflammatory antiviral responses. Nat. Immunol..

[65] Pearce E.J., Everts B. (2015). Dendritic cell metabolism. Nat. Rev. Immunol..

[66] Liu Z., Wang H., Li Z., Dress R.J., Zhu Y., Zhang S., de Feo D., Kong W.T., Cai P., Shin A. (2023). Dendritic cell type 3 arises from Ly6C+ monocyte-dendritic cell progenitors. Immunity.

[67] Böttcher J.P., Reis e Sousa C. (2018). The Role of Type 1 Conventional Dendritic Cells in Cancer Immunity. Trends Cancer.

[68] Kvedaraite E., Ginhoux F. (2022). Human dendritic cells in cancer. Sci. Immunol..

[69] Oh S.A., Wu D.-C., Cheung J., Navarro A., Xiong H., Cubas R., Totpal K., Chiu H., Wu Y., Comps-Agrar L. (2020). PD-L1 expression by dendritic cells is a key regulator of T-cell immunity in cancer. Nat. Cancer.

[70] Benteyn D., Heirman C., Bonehill A., Thielemans K., Breckpot K. (2015). mRNA-based dendritic cell vaccines. Expert Rev. Vaccines.

[71] Bordon Y. (2016). Dendritic cells: Sorting, sorted!. Nat. Rev. Immunol..

[72] Leone D.A., Rees A.J., Kain R. (2018). Dendritic cells and routing cargo into exosomes. Immunol. Cell Biol..

[73] Russo E., Teijeira A., Vaahtomeri K., Willrodt A.-H., Bloch J.S., Nitschké M., Santambrogio L., Kerjaschki D., Sixt M., Halin C. (2016). Intralymphatic CCL21 Promotes Tissue Egress of Dendritic Cells through Afferent Lymphatic Vessels. Cell Rep..

[74] Malinverno C., Corallino S., Giavazzi F., Bergert M., Li Q., Leoni M., Disanza A., Frittoli E., Oldani A., Martini E. (2017). Endocytic reawakening of motility in jammed epithelia. Nat. Mater..

[75] Schreibelt G., Klinkenberg L.J.J., Cruz L.J., Tacken P.J., Tel J., Kreutz M., Adema G.J., Brown G.D., Figdor C.G., de Vries I.J.M. (2012). The C-type lectin receptor CLEC9A mediates antigen uptake and (cross-)presentation by human blood BDCA3+ myeloid dendritic cells. Blood.

[76] Dos Santos Á., Hadjivasiliou A., Ossa F., Lim N.K., Turgut A., Taylor M.E., Drickamer K. (2017). Oligomerization domains in the glycan-binding receptors DC-SIGN and DC-SIGNR: Sequence variation and stability differences. Protein Sci. A Publ. Protein Soc..

[77] Jarvis C.M., Zwick D.B., Grim J.C., Alam M.M., Prost L.R., Gardiner J.C., Park S., Zimdars L.L., Sherer N.M., Kiessling L.L. (2019). Antigen structure affects cellular routing through DC-SIGN. Proc. Natl. Acad. Sci. USA.

[78] Chao P.-Z., Hsieh M.-S., Cheng C.-W., Hsu T.-J., Lin Y.-T., Lai C.-H., Liao C.-C., Chen W.-Y., Leung T.-K., Lee F.-P. (2015). Dendritic cells respond to nasopharygeal carcinoma cells through annexin A2-recognizing DC-SIGN. Oncotarget.

[79] Tanigaki K., Sundgren N., Khera A., Vongpatanasin W., Mineo C., Shaul P.W. (2015). Fcγ receptors and ligands and cardiovascular disease. Circ. Res..

[80] Proff J., Brey C.U., Ensser A., Holter W., Lehner M. (2018). Turning the tables on cytomegalovirus: Targeting viral Fc receptors by CARs containing mutated CH2-CH3 IgG spacer domains. J. Transl. Med..

[81] He H.-Q., Ye R.D. (2017). The Formyl Peptide Receptors: Diversity of Ligands and Mechanism for Recognition. Molecules.

[82] Unanue E.R., Turk V., Neefjes J. (2016). Variations in MHC Class II Antigen Processing and Presentation in Health and Disease. Annu. Rev. Immunol..

[83] Balasubbramanian D., Gelston C.A.L., Mitchell B.M., Chatterjee P. (2017). Toll-like receptor activation, vascular endothelial function, and hypertensive disorders of pregnancy. Pharmacol. Res..

[84] Baratin M., Foray C., Demaria O., Habbeddine M., Pollet E., Maurizio J., Verthuy C., Davanture S., Azukizawa H., Flores-Langarica A. (2015). Homeostatic NF-κB Signaling in Steady-State Migratory Dendritic Cells Regulates Immune Homeostasis and Tolerance. Immunity.

[85] Mann M., Mehta A., Zhao J.L., Lee K., Marinov G.K., Garcia-Flores Y., Lu L.-F., Rudensky A.Y., Baltimore D. (2017). An NF-κB-microRNA regulatory network tunes macrophage inflammatory responses. Nat. Commun..

[86] Vidya M.K., Kumar V.G., Sejian V., Bagath M., Krishnan G., Bhatta R. (2018). Toll-like receptors: Significance, ligands, signaling pathways, and functions in mammals. Int. Rev. Immunol..

[87] Feng Y., Mu R., Wang Z., Xing P., Zhang J., Dong L., Wang C. (2019). A toll-like receptor agonist mimicking microbial signal to generate tumor-suppressive macrophages. Nat. Commun..

[88] Kambayashi T., Laufer T.M. (2014). Atypical MHC class II-expressing antigen-presenting cells: Can anything replace a dendritic cell?. Nat. Rev. Immunol..

[89] Burrows S.R., Rossjohn J., McCluskey J. (2006). Have we cut ourselves too short in mapping CTL epitopes?. Trends Immunol..

[90] Roche P.A., Furuta K. (2015). The ins and outs of MHC class II-mediated antigen processing and presentation. Nat. Rev. Immunol..

[91] Blum J.S., Wearsch P.A., Cresswell P. (2013). Pathways of antigen processing. Annu. Rev. Immunol..

[92] Rossjohn J., Gras S., Miles J.J., Turner S.J., Godfrey D.I., McCluskey J. (2015). T cell antigen receptor recognition of antigen-presenting molecules. Annu. Rev. Immunol..

[93] Marigo I., Zilio S., Desantis G., Mlecnik B., Agnellini A.H.R., Ugel S., Sasso M.S., Qualls J.E., Kratochvill F., Zanovello P. (2016). T Cell Cancer Therapy Requires CD40-CD40L Activation of Tumor Necrosis Factor and Inducible Nitric-Oxide-Synthase-Producing Dendritic Cells. Cancer Cell.

[94] Liu Z., Ravindranathan R., Kalinski P., Guo Z.S., Bartlett D.L. (2017). Rational combination of oncolytic vaccinia virus and PD-L1 blockade works synergistically to enhance therapeutic efficacy. Nat. Commun..

[95] Buchholz V.R., Schumacher T.N.M., Busch D.H. (2016). T Cell Fate at the Single-Cell Level. Annu. Rev. Immunol..

[96] Wucherpfennig K.W., Gagnon E., Call M.J., Huseby E.S., Call M.E. (2010). Structural biology of the T-cell receptor: Insights into receptor assembly, ligand recognition, and initiation of signaling. Cold Spring Harb. Perspect. Biol..

[97] Moura Rosa P., Gopalakrishnan N., Ibrahim H., Haug M., Halaas Ø. (2016). The intercell dynamics of T cells and dendritic cells in a lymph node-on-a-chip flow device. Lab. A Chip.

[98] Liu X., Kwon H., Li Z., Fu Y.-X. (2017). Is CD47 an innate immune checkpoint for tumor evasion?. J. Hematol. Oncol..

[99] Nair-Gupta P., Baccarini A., Tung N., Seyffer F., Florey O., Huang Y., Banerjee M., Overholtzer M., Roche P.A., Tampé R. (2014). TLR signals induce phagosomal MHC-I delivery from the endosomal recycling compartment to allow cross-presentation. Cell.

[100] Steinman R.M., Banchereau J. (2007). Taking dendritic cells into medicine. Nature.

[101] Dauer M., Schad K., Herten J., Junkmann J., Bauer C., Kiefl R., Endres S., Eigler A. (2005). FastDC derived from human monocytes within 48 h effectively prime tumor antigen-specific cytotoxic T cells. J. Immunol. Methods.

[102] Mohty M., Vialle-Castellano A., Nunes J.A., Isnardon D., Olive D., Gaugler B. (2003). IFN-alpha skews monocyte differentiation into Toll-like receptor 7-expressing dendritic cells with potent functional activities. J. Immunol..

[103] Obleukhova I., Kiryishina N., Falaleeva S., Lopatnikova J., Kurilin V., Kozlov V., Vitsin A., Cherkasov A., Kulikova E., Sennikov S. (2018). Use of antigen-primed dendritic cells for inducing antitumor immune responses in vitro in patients with non-small cell lung cancer. Oncol. Lett..

[104] Breckpot K., Corthals J., Bonehill A., Michiels A., Tuyaerts S., Aerts C., Heirman C., Thielemans K. (2005). Dendritic cells differentiated in the presence of IFN-{beta} and IL-3 are potent inducers of an antigen-specific CD8+ T cell response. J. Leukoc. Biol..

[105] Tkach M., Kowal J., Zucchetti A.E., Enserink L., Jouve M., Lankar D., Saitakis M., Martin-Jaular L., Théry C. (2017). Qualitative differences in T-cell activation by dendritic cell-derived extracellular vesicle subtypes. EMBO J..

[106] Pitt J.M., André F., Amigorena S., Soria J.-C., Eggermont A., Kroemer G., Zitvogel L. (2016). Dendritic cell-derived exosomes for cancer therapy. J. Clin. Investig..

[107] Wei G., Jie Y., Haibo L., Chaoneng W., Dong H., Jianbing Z., Junjie G., Leilei M., Hongtao S., Yunzeng Z. (2017). Dendritic cells derived exosomes migration to spleen and induction of inflammation are regulated by CCR7. Sci. Rep..

[108] Oshita C., Takikawa M., Kume A., Miyata H., Ashizawa T., Iizuka A., Kiyohara Y., Yoshikawa S., Tanosaki R., Yamazaki N. (2012). Dendritic cell-based vaccination in metastatic melanoma patients: Phase II clinical trial. Oncol. Rep..

[109] Sennikov S.V., Shevchenko J.A., Kurilin V.V., Khantakova J.N., Lopatnikova J.A., Gavrilova E.V., Maksyutov R.A., Bakulina A.Y., Sidorov S.V., Khristin A.A. (2016). Induction of an antitumor response using dendritic cells transfected with DNA constructs encoding the HLA-A*02:01-restricted epitopes of tumor-associated antigens in culture of mononuclear cells of breast cancer patients. Immunol. Res..

[110] Shevchenko J.A., Lopatnikova J.A., Khantakova J.N., Silkov A.N., Kuznetsova M.S., Kurilin V.V., Maksyutov A.Z., Sennikov S.V. (2022). In Vitro Model of Suppression of the Alloantigen Response by Tolerogenic Dendritic Cells Transfected with Personalized DNA Constructs Encoding HLA Epitopes. Front. Biosci..

[111] Anassi E., Ndefo U.A. (2011). Sipuleucel-T (provenge) injection: The first immunotherapy agent (vaccine) for hormone-refractory prostate cancer. P T A Peer-Rev. J. Formul. Manag..

[112] Cheever M.A., Higano C.S. (2011). PROVENGE (Sipuleucel-T) in prostate cancer: The first FDA-approved therapeutic cancer vaccine. Clin. Cancer Res. Off. J. Am. Assoc. Cancer Res..

[113] Perez C.R., de Palma M. (2019). Engineering dendritic cell vaccines to improve cancer immunotherapy. Nat. Commun..

[114] Kurilin V., Kulikova E., Shevchenko J., Lopatnikova J., Obleukhova I., Khantakova J., Maksyutov A., Kuznetsova M., Khristin A., Kiryshina N. (2022). Dendritic cells transfected with a polyepitope DNA construct stimulate an antitumor cytotoxic response in various tumors. Mol. Clin. Oncol..

[115] Liau L.M., Prins R.M., Kiertscher S.M., Odesa S.K., Kremen T.J., Giovannone A.J., Lin J.-W., Chute D.J., Mischel P.S., Cloughesy T.F. (2005). Dendritic cell vaccination in glioblastoma patients induces systemic and intracranial T-cell responses modulated by the local central nervous system tumor microenvironment. Clin. Cancer Res. Off. J. Am. Assoc. Cancer Res..

[116] Castiello L., Sabatino M., Zhao Y., Tumaini B., Ren J., Ping J., Wang E., Wood L.V., Marincola F.M., Puri R.K. (2013). Quality controls in cellular immunotherapies: Rapid assessment of clinical grade dendritic cells by gene expression profiling. Mol. Ther. J. Am. Soc. Gene Ther..

[117] Turnis M.E., Rooney C.M. (2010). Enhancement of dendritic cells as vaccines for cancer. Immunotherapy.

[118] Li Y., Xu J., Zou H., Wang C. (2010). 1-MT enhances potency of tumor cell lysate-pulsed dendritic cells against pancreatic adenocarcinoma by downregulating the percentage of Tregs. J. Huazhong Univ. Sci. Technol. Med. Sci..

[119] Koido S., Homma S., Okamoto M., Namiki Y., Takakura K., Uchiyama K., Kajihara M., Arihiro S., Imazu H., Arakawa H. (2013). Fusions between dendritic cells and whole tumor cells as anticancer vaccines. Oncoimmunology.

[120] Chi H., Hao Y., Wang X., Tang L., Deng Y., Chen X., Gao F., Sha O., Jin G. (2022). A Therapeutic Whole-Tumor-Cell Vaccine Covalently Conjugated with a TLR7 Agonist. Cells.

[121] Aguilera R., Saffie C., van Tittarelli A., González F.E., Ramírez M., Reyes D., Pereda C., Hevia D., García T., Salazar L. (2011). Heat-shock induction of tumor-derived danger signals mediates rapid monocyte differentiation into clinically effective dendritic cells. Clin. Cancer Res. Off. J. Am. Assoc. Cancer Res..

[122] Flores I., Hevia D., Tittarelli A., Soto D., Rojas-Sepúlveda D., Pereda C., Tempio F., Fuentes C., Falcón-Beas C., Gatica J. (2019). Dendritic Cells Loaded with Heat Shock-Conditioned Ovarian Epithelial Carcinoma Cell Lysates Elicit T Cell-Dependent Antitumor Immune Responses In Vitro. J. Immunol. Res..

[123] Gleisner M.A., Pereda C., Tittarelli A., Navarrete M., Fuentes C., Ávalos I., Tempio F., Araya J.P., Becker M.I., González F.E. (2020). A heat-shocked melanoma cell lysate vaccine enhances tumor infiltration by prototypic effector T cells inhibiting tumor growth. J. Immunother. Cancer.

[124] Liu L.N., Shivakumar R., Allen C., Fratantoni J.C. (2008). Delivery of whole tumor lysate into dendritic cells for cancer vaccination. Methods Mol. Biol..

[125] Dong B., Dai G., Xu L., Zhang Y., Ling L., Sun L., Lv J. (2014). Tumor cell lysate induces the immunosuppression and apoptosis of mouse immunocytes. Mol. Med. Rep..

[126] Delirezh N., Moazzeni S.M., Shokri F., Shokrgozar M.A., Atri M., Kokhaei P. (2009). Autologous dendritic cells loaded with apoptotic tumor cells induce T cell-mediated immune responses against breast cancer in vitro. Cell. Immunol..

[127] Shevchenko J.A., Khristin A.A., Kurilin V.V., Kuznetsova M.S., Blinova D.D., Starostina N.M., Sidorov S.V., Sennikov S.V. (2020). Autologous dendritic cells and activated cytotoxic T-cells as combination therapy for breast cancer. Oncol. Rep..

[128] Berzofsky J.A., Terabe M., Oh S., Belyakov I.M., Ahlers J.D., Janik J.E., Morris J.C. (2004). Progress on new vaccine strategies for the immunotherapy and prevention of cancer. J. Clin. Investig..

[129] Viehl C.T., Tanaka Y., Chen T., Frey D.M., Tran A., Fleming T.P., Eberlein T.J., Goedegebuure P.S. (2005). Tat mammaglobin fusion protein transduced dendritic cells stimulate mammaglobin-specific CD4 and CD8 T cells. Breast Cancer Res. Treat..

[130] Yang H., Cho N.-H., Seong S.-Y. (2009). The Tat-conjugated N-terminal region of mucin antigen 1 (MUC1) induces protective immunity against MUC1-expressing tumours. Clin. Exp. Immunol..

[131] Zhou Y., Bosch M.L., Salgaller M.L. (2002). Current methods for loading dendritic cells with tumor antigen for the induction of antitumor immunity. J. Immunother..

[132] Pol J., Bloy N., Buqué A., Eggermont A., Cremer I., Sautès-Fridman C., Galon J., Tartour E., Zitvogel L., Kroemer G. (2015). Trial Watch: Peptide-based anticancer vaccines. Oncoimmunology.

[133] Knutson K.L., Schiffman K., Cheever M.A., Disis M.L. (2002). Immunization of cancer patients with a HER-2/neu, HLA-A2 peptide, p369–377, results in short-lived peptide-specific immunity. Clin. Cancer Res. Off. J. Am. Assoc. Cancer Res..

[134] Mittendorf E.A., Wu Y., Scaltriti M., Meric-Bernstam F., Hunt K.K., Dawood S., Esteva F.J., Buzdar A.U., Chen H., Eksambi S. (2009). Loss of HER2 amplification following trastuzumab-based neoadjuvant systemic therapy and survival outcomes. Clin. Cancer Res. Off. J. Am. Assoc. Cancer Res..

[135] Kirk C.J., Mulé J.J. (2000). Gene-modified dendritic cells for use in tumor vaccines. Hum. Gene Ther..

[136] Nakamura M., Iwahashi M., Nakamori M., Ueda K., Ojima T., Naka T., Ishida K., Yamaue H. (2005). Dendritic cells transduced with tumor-associated antigen gene elicit potent therapeutic antitumor immunity: Comparison with immunodominant peptide-pulsed DCs. Oncology.

[137] Marchini C., Kalogris C., Garulli C., Pietrella L., Gabrielli F., Curcio C., Quaglino E., Cavallo F., Amici A. (2013). Tailoring DNA Vaccines: Designing Strategies Against HER2-Positive Cancers. Front. Oncol..

[138] Palucka K., Ueno H., Banchereau J. (2011). Recent developments in cancer vaccines. J. Immunol..

[139] Mishinov S.V., Budnik A.Y., Stupak V.V., Leplina O.Y., Tyrinova T.V., Ostanin A.A., Chernykh E.R. (2020). Autologous and Pooled Tumor Lysates in Combined Immunotherapy of Patients with Glioblastoma. Sovrem. Tekhnologii V Meditsine.

[140] Markov O.V., Mironova N.L., Sennikov S.V., Vlassov V.V., Zenkova M.A. (2015). Prophylactic Dendritic Cell-Based Vaccines Efficiently Inhibit Metastases in Murine Metastatic Melanoma. PLoS ONE.

[141] Luo D., Saltzman W.M. (2000). Synthetic DNA delivery systems. Nat. Biotechnol..

[142] Lundqvist A., Noffz G., Pavlenko M., Saebøe-Larssen S., Fong T., Maitland N., Pisa P. (2002). Nonviral and viral gene transfer into different subsets of human dendritic cells yield comparable efficiency of transfection. J. Immunother..

[143] Grünebach F., Müller M.R., Brossart P. (2005). New developments in dendritic cell-based vaccinations: RNA translated into clinics. Cancer Immunol. Immunother..

[144] Najafi S., Mortezaee K. (2023). Advances in dendritic cell vaccination therapy of cancer. Biomed. Pharmacother. Biomed. Pharmacother..

[145] Raïch-Regué D., Glancy M., Thomson A.W. (2014). Regulatory dendritic cell therapy: From rodents to clinical application. Immunol. Lett..

[146] MacNabb B.W., Chen X., Tumuluru S., Godfrey J., Kasal D.N., Yu J., Jongsma M.L.M., Spaapen R.M., Kline D.E., Kline J. (2022). Dendritic cells can prime anti-tumor CD8+ T cell responses through major histocompatibility complex cross-dressing. Immunity.

[147] Chen J., Guo X.-Z., Li H.-Y., Wang D., Shao X. (2015). Comparison of cytotoxic T lymphocyte responses against pancreatic cancer induced by dendritic cells transfected with total tumor RNA and fusion hybrided with tumor cell. Exp. Biol. Med..

[148] van Nuffel A.M.T., Corthals J., Neyns B., Heirman C., Thielemans K., Bonehill A. (2010). Immunotherapy of cancer with dendritic cells loaded with tumor antigens and activated through mRNA electroporation. Methods Mol. Biol..

[149] Garg A.D., Coulie P.G., van den Eynde B.J., Agostinis P. (2017). Integrating Next-Generation Dendritic Cell Vaccines into the Current Cancer Immunotherapy Landscape. Trends Immunol..

[150] Schuurhuis D.H., Verdijk P., Schreibelt G., Aarntzen E.H.J.G., Scharenborg N., de Boer A., van de Rakt M.W.M.M., Kerkhoff M., Gerritsen M.-J.P., Eijckeler F. (2009). In situ expression of tumor antigens by messenger RNA-electroporated dendritic cells in lymph nodes of melanoma patients. Cancer Res..

[151] Bedrosian I., Mick R., Xu S., Nisenbaum H., Faries M., Zhang P., Cohen P.A., Koski G., Czerniecki B.J. (2003). Intranodal administration of peptide-pulsed mature dendritic cell vaccines results in superior CD8+ T-cell function in melanoma patients. J. Clin. Oncol. Off. J. Am. Soc. Clin. Oncol..

[152] Fong L., Brockstedt D., Benike C., Wu L., Engleman E.G. (2001). Dendritic cells injected via different routes induce immunity in cancer patients. J. Immunol..

[153] Rodríguez-Ruiz M.E., Perez-Gracia J.L., Rodríguez I., Alfaro C., Oñate C., Pérez G., Gil-Bazo I., Benito A., Inogés S., López-Diaz de Cerio A. (2018). Combined immunotherapy encompassing intratumoral poly-ICLC, dendritic-cell vaccination and radiotherapy in advanced cancer patients. Ann. Oncol. Off. J. Eur. Soc. Med. Oncol..

[154] Amberger D.C., Schmetzer H.M. (2020). Dendritic Cells of Leukemic Origin: Specialized Antigen-Presenting Cells as Potential Treatment Tools for Patients with Myeloid Leukemia. Transfus. Med. Hemotherapy.

[155] van Lint S., Wilgenhof S., Heirman C., Corthals J., Breckpot K., Bonehill A., Neyns B., Thielemans K. (2014). Optimized dendritic cell-based immunotherapy for melanoma: The TriMix-formula. Cancer Immunol. Immunother..

[156] Bonehill A., van Nuffel A.M.T., Corthals J., Tuyaerts S., Heirman C., François V., Colau D., van der Bruggen P., Neyns B., Thielemans K. (2009). Single-step antigen loading and activation of dendritic cells by mRNA electroporation for the purpose of therapeutic vaccination in melanoma patients. Clin. Cancer Res. Off. J. Am. Assoc. Cancer Res..

[157] Anguille S., Smits E.L., Lion E., Tendeloo V.F., Berneman Z.N. (2014). Clinical use of dendritic cells for cancer therapy. Lancet. Oncol..

[158] van Lint S., Renmans D., Broos K., Dewitte H., Lentacker I., Heirman C., Breckpot K., Thielemans K. (2015). The ReNAissanCe of mRNA-based cancer therapy. Expert Rev. Vaccines.

[159] Rosa F.F., Pires C.F., Kurochkin I., Ferreira A.G., Gomes A.M., Palma L.G., Shaiv K., Solanas L., Azenha C., Papatsenko D. (2018). Direct reprogramming of fibroblasts into antigen-presenting dendritic cells. Sci. Immunol..

[160] Okada N., Mori N., Koretomo R., Okada Y., Nakayama T., Yoshie O., Mizuguchi H., Hayakawa T., Nakagawa S., Mayumi T. (2005). Augmentation of the migratory ability of DC-based vaccine into regional lymph nodes by efficient CCR7 gene transduction. Gene Ther..

[161] Dong H., Zhu G., Tamada K., Chen L. (1999). B7-H1, a third member of the B7 family, co-stimulates T-cell proliferation and interleukin-10 secretion. Nat. Med..

[162] van der Waart A.B., Fredrix H., van der Voort R., Schaap N., Hobo W., Dolstra H. (2015). siRNA silencing of PD-1 ligands on dendritic cell vaccines boosts the expansion of minor histocompatibility antigen-specific CD8^+^ T cells in NOD/SCID/IL2Rg(null) mice. Cancer Immunol. Immunother..

[163] Hurwitz A.A., Watkins S.K. (2012). Immune suppression in the tumor microenvironment: A role for dendritic cell-mediated tolerization of T cells. Cancer Immunol. Immunother..

[164] Kuznetsova M., Lopatnikova J., Shevchenko J., Silkov A., Maksyutov A., Sennikov S. (2019). Cytotoxic Activity and Memory T Cell Subset Distribution of in vitro-Stimulated CD8+ T Cells Specific for HER2/neu Epitopes. Front. Immunol..

[165] Dong H., Strome S.E., Salomao D.R., Tamura H., Hirano F., Flies D.B., Roche P.C., Lu J., Zhu G., Tamada K. (2002). Tumor-associated B7-H1 promotes T-cell apoptosis: A potential mechanism of immune evasion. Nat. Med..

[166] Curiel T.J., Wei S., Dong H., Alvarez X., Cheng P., Mottram P., Krzysiek R., Knutson K.L., Daniel B., Zimmermann M.C. (2003). Blockade of B7-H1 improves myeloid dendritic cell-mediated antitumor immunity. Nat. Med..

[167] Squadrito M.L., Cianciaruso C., Hansen S.K., de Palma M. (2018). EVIR: Chimeric receptors that enhance dendritic cell cross-dressing with tumor antigens. Nat. Methods.

[168] Hobo W., Maas F., Adisty N., de Witte T., Schaap N., van der Voort R., Dolstra H. (2010). siRNA silencing of PD-L1 and PD-L2 on dendritic cells augments expansion and function of minor histocompatibility antigen-specific CD8+ T cells. Blood.

[169] Kuznetsova M., Lopatnikova J., Khantakova J., Maksyutov R., Maksyutov A., Sennikov S. (2017). Generation of populations of antigen-specific cytotoxic T cells using DCs transfected with DNA construct encoding HER2/neu tumor antigen epitopes. BMC Immunol..

[170] Constantino J., Gomes C., Falcão A., Neves B.M., Cruz M.T. (2017). Dendritic cell-based immunotherapy: A basic review and recent advances. Immunol. Res..

[171] Ojima T., Iwahashi M., Nakamura M., Matsuda K., Nakamori M., Ueda K., Naka T., Ishida K., Primus F.J., Yamaue H. (2007). Successful cancer vaccine therapy for carcinoembryonic antigen (CEA)-expressing colon cancer using genetically modified dendritic cells that express CEA and T helper-type 1 cytokines in CEA transgenic mice. Int. J. Cancer.

[172] Ojima T., Iwahashi M., Nakamura M., Matsuda K., Nakamori M., Ueda K., Naka T., Katsuda M., Miyazawa M., Iida T. (2008). Streptococcal preparation OK-432 promotes the capacity of dendritic cells (DCs) to prime carcinoembryonic antigen (CEA)-specific cytotoxic T lymphocyte responses induced with genetically modified DCs that express CEA. Int. J. Oncol..

[173] Miyazawa M., Iwahashi M., Ojima T., Katsuda M., Nakamura M., Nakamori M., Ueda K., Naka T., Hayata K., Iida T. (2011). Dendritic cells adenovirally-transduced with full-length mesothelin cDNA elicit mesothelin-specific cytotoxicity against pancreatic cancer cell lines in vitro. Cancer Lett..

